# A Lymph Node Count-Based AJCC Staging System Facilitates a More Accurate Prediction of the Prognosis of Patients With Endometrial Cancer

**DOI:** 10.3389/fonc.2021.641962

**Published:** 2021-03-03

**Authors:** Xinlong Huo, Shufang Wang

**Affiliations:** ^1^Department of Oncology, The First Hospital of Qinhuangdao City, Qinhuangdao, China; ^2^Department of Obstetrics and Gynecology, Maternal and Child Health Care Hospital of Qinhuangdao, Qinhuangdao, China

**Keywords:** endometrial cancer, AJCC, overall survival, SEER, lymph node

## Abstract

**Purpose:**

Both the International Federation of Gynecology and Obstetrics (FIGO) and the American Joint Committee on Cancer (AJCC) staging system for endometrial cancer (EC) defined the N category by the location of metastatic lymph nodes (LNs) rather than the metastatic LN count. We aimed to compare the accuracy of the AJCC staging system and the LN count-based staging system.

**Patients and Methods:**

EC patients were selected from the Surveillance, Epidemiology and End Results (SEER) database between 2004 and 2016. Patients’ characteristics were collected, including age, race, marital status, histological type, grade, therapeutic measures, the number of metastatic LNs, the number of dissected LNs, vital status, and survival in months. Overall survival (OS) was analyzed by the Kaplan–Meier (KM) method and the concordance index (C-index) was used to compare the prognostic value of the AJCC staging system and the LN count-based staging system.

**Results:**

We identified 4,276 EC cases from the SEER database, including 2,693 patients with stage IIIC1 and 1,583 patients with stage IIIC2. Multivariate analyses showed that independent prognostic factors for patients with stage IIIC1 included age, race, marital status, grade, histology, chemotherapy, and radiotherapy. Independent prognostic factors for patients with stage IIIC2 included age, marital status, grade, histology, chemotherapy, and radiotherapy. The C-index of the AJCC staging system and the LN count-based staging system were 0.483 and 0.617, respectively. At least six LNs should be dissected to ensure the accuracy of the LN count-based staging system.

**Conclusion:**

A modified AJCC staging system based on the count of metastatic LNs might be superior to the current AJCC staging system, which still had room for improvement and further refinements were required. For accurate staging, we recommended that at least six LNs should be examined in the modified AJCC staging system.

## Introduction

Endometrial cancer (EC) is a malignant epithelial carcinoma that originates from the inner lining of the uterus (1). It represents one of the most common cancer types of the female reproductive system, accounting for approximately 4–6% of all cancers in women (2, 3). Treatment strategies depend on the stage of EC. There’s still a controversial in the role of lymph node dissection for patients with early-stage EC (4). For EC patients with stage IIIC, however, the standard approach is a total hysterectomy and bilateral salpingo-oophorectomy with lymph nodes assessment based on the National Comprehensive Cancer Network (NCCN) guidelines (5). The prognosis of EC also differed according to the stage (6). Primary adverse prognostic factors for EC patients include advanced pathological stage, lymph node (LN) involvement, lymphovascular space invasion (LVSI), greater tumor size, deeper level of myometrial invasion, high-risk histological subtypes, and the formation of aneuploidy (7–12). Among patients with EC without distant metastasis, the LN metastasis rate is up to 13–29% (13–16). The 5-year survival rate of patients with LN metastasis is approximately 60%, while that of those without LN metastasis could be over 80% (17). Therefore, EC with LN metastasis should be given more attention.

The most commonly used staging system for EC is the 2009 International Federation of Gynecology and Obstetrics (FIGO) staging system (18). Based on the FIGO system (2009), the American Joint Committee on Cancer (AJCC) released the 8th edition of the tumor-node-metastasis (TNM) staging system in 2016 (19). Both the FIGO and AJCC staging system share the same N classification: an N1 category (metastasis to pelvic LNs) and an N2 category (metastasis to para‐aortic LNs with or without metastasis to pelvic LNs). Patients with stage IIIC2 EC (T1-3N2M0) indeed have a higher mortality rate than those with stage IIIC1 (T1-3N1M0) (20). According to the convention of the AJCC staging system, however, the N classification is usually determined by the number of metastatic LNs rather than the location. For example, the N classification was divided into the N1 category (metastatic LNs along the cystic duct, common bile duct, hepatic artery, and/or portal vein) and N2 category (metastatic LNs to periaortic, pericaval, superior mesenteric artery, and/or celiac artery) based on the location of metastatic LNs in the 7th edition of the AJCC staging system for gallbladder cancer (21). In the 8th edition of the AJCC staging system for gallbladder cancer, the N classification had been revised as follows: the N1 category includes 1–3 metastatic LNs and the N2 category includes ≥4 metastatic LNs (22). Therefore, there is a need to evaluate whether the N classification could also be divided by the number of metastatic LNs in the next edition of the AJCC staging system for EC.

In the present study, we attempted to introduce the number of metastatic LNs instead of the location of metastatic LNs in the AJCC staging system using the Surveillance, Epidemiology and End Results (SEER) database, with the goal to more accurately stage EC.

## Materials and Methods

### Ethics Statement

This study was approved by the Institutional Review Board of Maternal and Child Health Care Hospital of Qinhuangdao. The informed consent of all patients in the SEER database has been obtained before the publication of the database by the National Institutes of Health (NIH) Ethics Office.

### Patients

Patients with EC included in the SEER database between 2004 and 2016 were selected for this study. Our inclusion criteria for this study were: 1) 18 years of age or older; 2) first primary tumor; 3) histologically diagnosed as EC; 4) definite T category (T1-3) and N category (N1-2) according to the 8th edition of the AJCC staging system for EC; 5) the number of metastatic LNs is definite; 6) no distant metastases; 7) definite therapeutic measures (including surgery, chemotherapy, and radiotherapy); 8) available follow-up data. The follow-up period ranged from 0 to 83 months with a median follow-up time of 30 months. Patients with EC included in the database before 2004 were excluded as, for those patients, the staging information was incomplete. To modify the AJCC staging system, patients without definite LN count were also excluded from the study.

We collected patient characteristics, including age, race, marital status, histological type, grade, therapeutic measures, the number of metastatic LNs, the number of dissected LNs, vital status, and survival months.

### Statistical Analysis

The basic characteristics of the entire cohort were compared using the chi-square test. We used Cox proportional-hazards regression analyses to identify independent predictors. Overall survival (OS) was defined as the primary endpoint and analyzed using the Kaplan–Meier (KM) method. The optimal number of dissected LNs was determined by the X-tile software (Yale University, Version 3.6.1). The Concordance index (C-index) was used to compare the prognostic value of the two staging systems (AJCC staging system and modified AJCC staging system) using the R package “survcomp.” A two-tailed p < 0.05 was defined as a statistically significant difference.

## Results

We identified 4,276 patients with EC from the SEER database, including 2,693 patients with stage IIIC1 and 1,583 patients with stage IIIC2 tumors. The characteristics of the cohort are summarized in **Table 1**. The median age was 63 years (ranging from 19 to 94). The majority of patients were White (3220, 75.3%), and approximately half of the patients (1982, 46.4%) were married at the time of diagnosis. We included 1,455 (34.0%) patients with well/moderately differentiated tumors (grade I + II), and 1,946 (45.5%) patients with poorly differentiated or undifferentiated tumors (grade III+IV). The main histological subtypes were endometrioid [2,326 patients (54.4%)] and serous [656 patients (15.3%)]. Nearly all patients (99.7%) underwent surgical operations, 3,315 (77.5%) patients received chemotherapy, and 2,259 (52.8%) patients received radiotherapy. The median number of dissected LNs was 16 (ranging from 1 to 88), and the median number of positive LNs was 2 (ranging from 1 to 66).

**Table 1:** Characteristics of stage IIIC patients with endometrial cancer.

Characteristics	Total (%)	Stage IIIC1 (%)	Stage IIIC2 (%)	P
**No. of patients**	4276	2693	1583	
**Age, years**				0.045
**18–60**	1,748 (40.9%)	1,132 (42.0%)	616 (38.9%)	
**>60**	2,528 (59.1%)	1,561 (58.0%)	967 (61.1%)	
**Race**				0.102
**White**	3,220 (75.3%)	2,057 (76.4%)	1,163 (73.5%)	
**Black**	560 (13.1%)	337 (12.5%)	223 (14.1%)	
**Others**	496 (11.6%)	299 (11.1%)	197 (12.4%)	
**Marital status**				0.739
**Married**	1,982 (46.4%)	1,243 (46.2%)	739 (46.7%)	
**Others**	2,294 (53.6%)	1,450 (53.8%)	844 (53.3%)	
**Grade**				<0.001
**I+II**	1,455 (34.0%)	998 (37.1%)	457 (28.9%)	
**III+IV**	1,946 (45.5%)	1,112 (41.3%)	834 (52.7%)	
**Others**	875 (20.5%)	583 (21.6%)	292 (18.4%)	
**Histology**				<0.001
**Endometrioid**	2326 (54.4%)	1,553 (57.7%)	773 (48.8%)	
**Serous**	656 (15.3%)	342 (12.7%)	314 (19.8%)	
**Others**	1,294 (30.3%)	798 (29.6%)	496 (31.4%)	
**Surgery**				0.914
**Yes**	4,263 (99.7%)	2,685 (99.7%)	1,578 (99.7%)	
**No/unknown**	13 (0.3%)	8 (0.3%)	5 (0.3%)	
**Chemotherapy**				0.010
**Yes**	3,315 (77.5%)	2,054 (76.3%)	1,261 (79.7%)	
**No/unknown**	961 (22.5%)	639 (23.7%)	322 (20.3%)	
**Radiotherapy**				0.002
**Yes**	2,259 (52.8%)	1,471 (54.6%)	788 (49.8%)	
**No/unknown**	2,017 (47.2%)	1,222 (45.4%)	795 (50.2%)	
No. of examined LNs*				
**Range (median)**	1–88 (16)	1–88 (15)	1–83 (19)	
**No. of positive LNs**				
**Range (median)**	1–66 (2)	1–29 (1)	1–66 (4)	

*24 cases missing.

To identify the possible predictive factors, we performed univariate and multivariate Cox proportional-hazards regression analyses. It was to be notated that surgical factor was excluded because only 0.3% of the cohort did not received surgery. Univariate analyses suggested that all the included variables were predictors of clinical outcomes (**Table 2**). Multivariate analyses revealed that independent prognostic factors for patients with stage IIIC1 included age, race, marital status, grade, histology, chemotherapy, and radiotherapy (**Table 3**). Independent prognostic factors for patients with stage IIIC2 included age, marital status, grade, histology, chemotherapy, and radiotherapy (**Table 3**).

**Table 2:** Univariate analysis for overall survival (OS) of stage IIIC patients with endometrial cancer.

Variables	Stage IIIC1		Stage IIIC2	
	HR (95% CI)	P	HR (95% CI)	P
**Age, yrs**				
**18–60**	Reference		Reference	
**>60**	2.017 (1.762–2.308)	<0.001	1.778 (1.513–2.090)	<0.001
**Race**				
**White**	Reference		Reference	
**Black**	1.688 (1.443–1.974)	<0.001	1.650 (1.372–1.985)	<0.001
**Others**	0.634 (0.497–0.808)	<0.001	0.842 (0.660–1.074)	0.166
**Marital status**				
**Married**	Reference		Reference	
**Others**	1.574 (1.387–1.785)	<0.001	1.549 (1.333–1.800)	<0.001
**Grade**				
**I+II**	Reference		Reference	
**III+IV**	3.414 (2.930–3.977)	<0.001	3.321 (2.706–4.077)	<0.001
**Others**	2.027 (1.676–2.452)	<0.001	2.170 (1.680–2.804)	<0.001
**Histology**				
**Endometrioid**	Reference		Reference	
**Serous**	2.534 (2.135–3.009)	<0.001	2.277 (1.881–2.757)	<0.001
**Others**	2.616 (2.290–2.988)	<0.001	2.131 (1.802–2.520)	<0.001
**Chemotherapy**				
**Yes**	Reference		Reference	
**No/unknown**	1.694 (1.503–1.911)	<0.001	1.701 (1.468–1.970)	<0.001
**Radiotherapy**				
**Yes**	Reference		Reference	
**No/unknown**	1.959 (1.729–2.219)	<0.001	1.753 (1.507–2.039)	<0.001

**Table 3:** Multivariate analysis for overall survival (OS) of stage IIIC patients with endometrial cancer.

Variables	Stage IIIC1		Stage IIIC2	
	HR (95% CI)	P	HR (95% CI)	P
**Age, yrs**				
**18–60**	Reference		Reference	
**>60**	1.579 (1.375–1.814)	<0.001	1.481 (1.255–1.748)	<0.001
**Race**				
**White**	Reference		Reference	
**Black**	1.310 (1.117–1.537)	0.001	1.156 (0.955–1.400)	0.136
**Others**	0.754 (0.590–0.963)	0.024	0.896 (0.700–1.147)	0.383
**Marital status**				
**Married**	Reference		Reference	
**Others**	1.303 (1.145–1.484)	<0.001	1.366 (1.171–1.593)	<0.001
**Grade**				
**I+II**	Reference		Reference	
**III+IV**	2.528 (2.143–2.983)	<0.001	2.555 (2.040–3.200)	<0.001
**Others**	1.809 (1.491–2.195)	<0.001	1.884 (1.452–2.446)	<0.001
**Histology**				
**Endometrioid**	Reference		Reference	
**Serous**	1.406 (1.167–1.694)	<0.001	1.394 (1.128–1.722)	<0.001
**Others**	1.883 (1.637–2.166)	<0.001	1.459 (1.217–1.749)	<0.001
**Chemotherapy**				
**Yes**	Reference		Reference	
**No/unknown**	1.446 (1.287–1.670)	<0.001	1.581 (1.350–1.853)	<0.001
**Radiotherapy**				
**Yes**	Reference		Reference	
**No/unknown**	1.526 (1.336–1.743)	<0.001	1.376 (1.170–1.619)	<0.001

**Figure 1:**
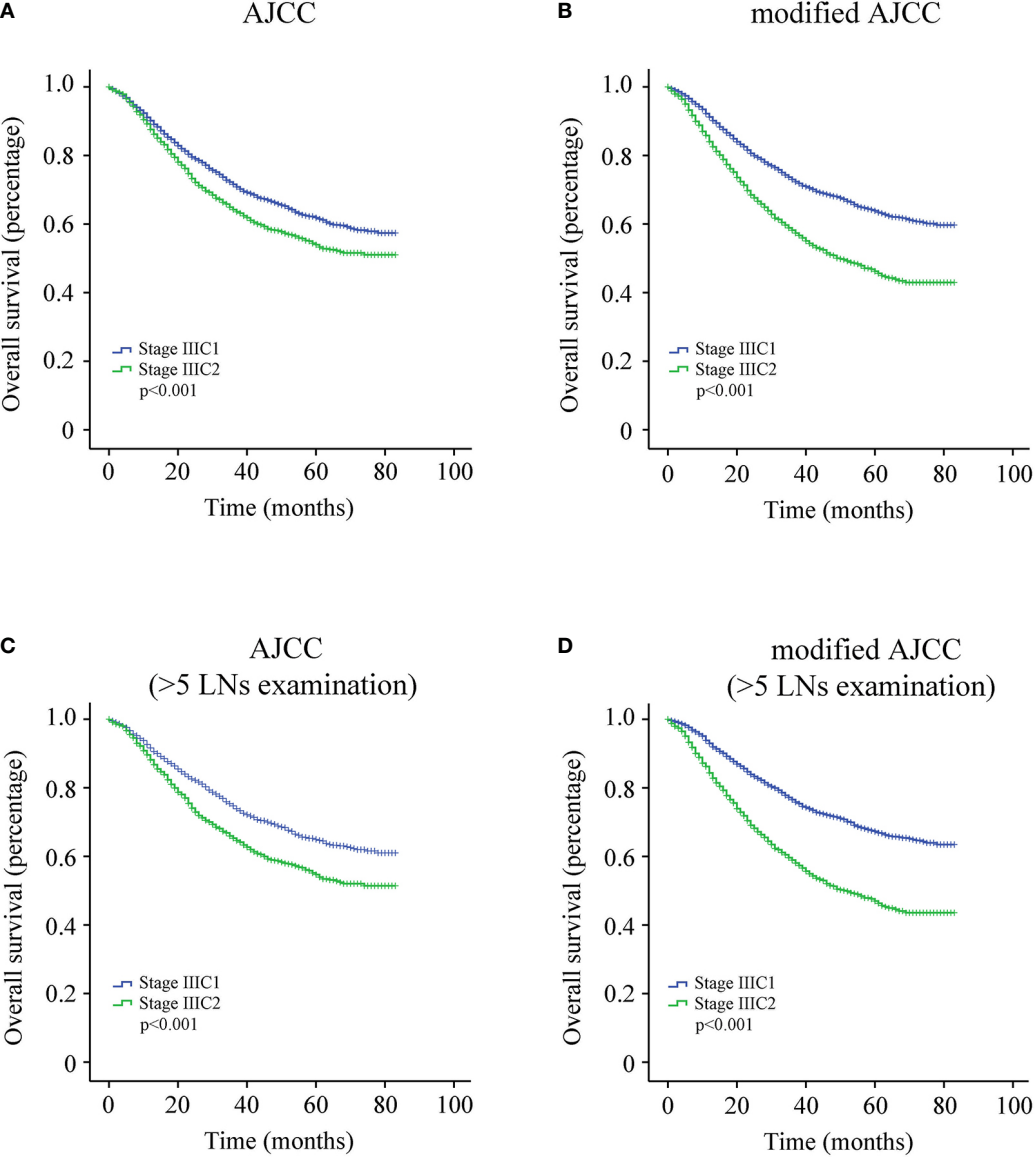
**(A)** Survival curve of entire cohort according to the American Joint Committee on Cancer (AJCC) staging system. **(B)** Survival curve of entire cohort according to the modified AJCC staging system. **(C)** Survival curve of endometrial cancer (EC) patients with more than five lymph nodes (LNs) resected according to the AJCC staging system. **(D)** Survival curve of EC patients with more than five LNs resected according to the modified AJCC staging system.

The KM survival analysis revealed that patients with stage IIIC1 had better clinical outcomes than those with stage IIIC2 (**Figure 1A**, p < 0.001). The overall 5-year survival rate of patients with stage IIIC1 was 61.8%, whereas that of patients with stage IIIC2 was 54.0%. The cohort was then divided into two groups according to the number of positive LNs: modified stage IIIC1 (1–3 positive LNs) and modified stage IIIC2 (four and more positive LNs). KM analysis showed that patients with modified stage IIIC1 had a better clinical outcome than those with modified stage IIIC2 (**Figure 1B**, p < 0.001). The overall 5-year survival rate of patients with modified stage IIIC1 was 63.8%, whereas that of patients with modified stage IIIC2 was 46.2%. The C-index of the AJCC staging system and the modified AJCC staging system were 0.483 [95% confidence interval (CI), 0.458–0.508] and 0.617 [95% CI, 0.592–0.642], respectively. This indicates that the modified AJCC staging system could distinguish the risk of death of EC better than the AJCC staging system.

**Figure 2:**
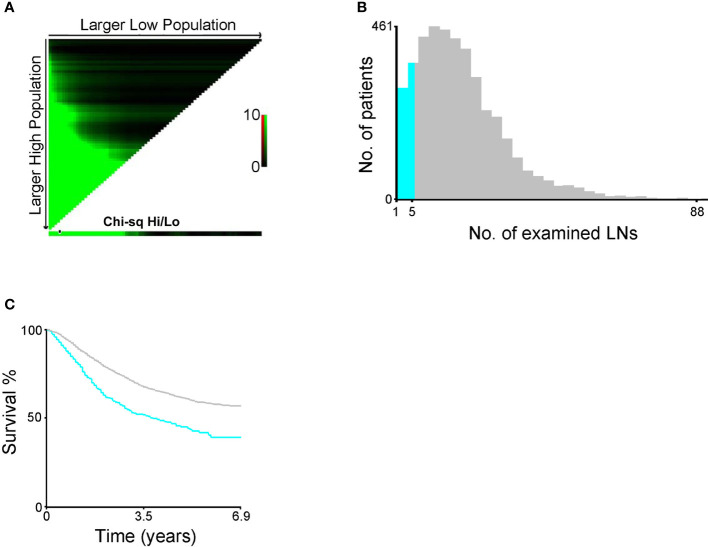
**(A)** X-tile plots (the black circle represented the optimal number. **(B)** The distribution of number of endometrial cancer (EC) patients according to the number of resected lymph nodes (LNs). **(C)** Survival curve of EC patients divided by the optimal number of resected LNs.

We further attempted to determine the optimal number of dissected LNs that is most beneficial for patients with EC using the X-tile software (Yale University, Version 3.6.1). Our data indicate that at least six (more than five) LNs should be dissected (**Figure 2**). Therefore, EC patients with more than five dissected LNs were identified for further KM analysis of 3,726 cases. Among these patients, the overall 5-year survival rate of patients with stage IIIC1 was 65.0%, whereas that of patients with stage IIIC2 was 54.7% (**Figure 1C**, p < 0.001). The overall 5-year survival rate of patients with modified stage IIIC1 was 67.3%, whereas that of patients with modified stage IIIC2 was 47.0% (**Figure 1D**, p < 0.001). The C-index scores of the AJCC staging system and the modified AJCC staging system were 0.520 (95% CI, 0.494-0.546) and 0.590 (95% CI, 0.563–0.617), respectively. The modified AJCC staging system also exhibited a superior prognostic power.

## Discussion

Using the SEER database, we found that a modified AJCC staging system based on the count of metastatic LNs might be superior to the current AJCC staging system, which still had room for improvement, and further refinements were required. For accurate staging, we recommend that at least six LNs should be examined for the modified AJCC staging system. To our best knowledge, this is the first study to introduce the number of metastatic LNs into the AJCC staging system. These findings could provide better treatment suggestion and management for patients with EC, and facilitate more accurate prediction of their prognosis in clinical practice.

In fact, there is still controversy regarding which patient groups require lymphadenectomy or which procedures (e.g., biopsies, sentinel LN mapping, pelvic lymphadenectomy alone or combined pelvic and para-aortic lymphadenectomy) should be used since the risk of LN metastasis varies in different patient groups (23–27). Sentinel LN mapping is the emerging concept for EC. According to the current literature, sentinel LN mapping is quite promising in the treatment of EC, which can reduce the occurrence of postoperative complications, including lymphedema and lymphocyst formation (28–30). The emergence of minimally invasive techniques could also further improves the quality of life (QoL) of patients with EC (31, 32). For patients with LN metastasis, however, lymphadenectomy was significantly associated with improved survival outcomes in various large population-based studies (33–35). Todo et al. (34) reported that combined pelvic and para-aortic lymphadenectomy could prolong the OS time of patients with intermediate/high risk of recurrence (stage III and IV were classified into the high- risk group). Havrilesky et al. (36) also believed that patients with stage IIIC would benefit from lymphadenectomy. Kikuchi et al. (37) reported that most EC patients with stage IIIC1 received pelvic lymphadenectomy alone while those with stage IIIC2 received combined pelvic and para-aortic lymphadenectomy (at least para-aortic LN sampling) at their institution. As a result, patients with stage IIIC1 did not achieve a remarkable survival advantage over those with stage IIIC2. This indicates that extensive LN dissection might be an appropriate treatment modality for patients with stage IIIC.

The optimal dissection range of LNs also remains controversial. The SEER data showed that the median number of resected LNs among all patients undergoing LN assessment (stage I–IV) was 7 and 12 during the time periods 1988–1992 and 1998–2001, respectively (38). Our study was also derived from the SEER database and showed that the median number of resected LNs among EC patients with stage IIIC was 16. The dissection range of LNs seemed to be extended over time. Compared with other studies, however, the dissection range of LNs from the SEER database was still relatively conservative. Fujimoto et al. (39) reported that the median number of resected LNs was 51 and 21 for stage IIIC1 and stage IIIC2, respectively. The SEPAL study from Japan showed that the median number of resected LNs was 34 in pelvic LN dissection group and 82 in the para‐aortic/pelvic LN dissection group (34). However, more radical LN dissection was not representative of better clinical outcomes. Extensive LN dissection also increased the risk of perioperative complications, including greater blood loss, ureteral or intestinal injury, and lymphedema. Although the median number of LNs resected has frequently been used as the cut-off value of the optimal dissection range of LNs, it was arbitrary and not practical because it varied from one study to another. It had been suggested that at least 10 pelvic LNs and five para-aortic LNs should be dissected, but this suggestion had not been well supported by scientific evidence (33). Our modified staging system based on the SEER database suggests that at least six LNs should be examined. Indeed, more prospective data are required to verify our results.

Additionally, Odagiri et al. (16) showed that 61.9% (26/42) of patients with stage IIIC exhibited metastasis to pelvic LNs alone (stage IIIC1) while 38.1% (16/42) exhibited metastasis to para-aortic LNs (with or without pelvic LNs, stage IIIC2). A single-center retrospective study also reported that a larger proportion of patients with LN metastasis were classified as stage IIIC2 rather than stage IIIC1 based on the AJCC staging system (40). These results are not consistent with our population-based findings that 37.0% (1,583/4,276) of patients were classified as stage IIIC1. The inconsistency may be due to the small sample size in these studies.

There are several limitations to the present study. First, the SEER data were retrospective, which has an inherent limitation by nature. Second, information about cancer recurrence and procedure-related complications are not recorded in the SEER database. Third, each cancer has different molecular and clinical characteristics. To define whether it is appropriate to introduce the LN count into the definition of the N category, we must determine if the LN count-based staging system has advantages over the LN location-based staging system. Nonetheless, we offered a possibility of improving the current staging system.

## Data Availability Statement

Publicly available datasets were analyzed in this study. This data can be found here: https://seer.cancer.gov.

## Ethics Statement

This study was approved by the Institutional Review Board of Maternal and Child Health Care Hospital of Qinhuangdao. The informed consent of all patients in the SEER database has been obtained before the publication of the database by the National Institutes of Health (NIH) Ethics Office.

## Author Contributions

SW made substantial contributions to the design of the study, carried out the analysis, interpreted the data. XH contributed to the review of previous literature, data discussion, and critically commented on the manuscript for scientific content. All authors contributed to the article and approved the submitted version.

## Conflict of Interest

The authors declare that the research was conducted in the absence of any commercial or financial relationships that could be construed as a potential conflict of interest.
